# A Rare Case of Shunt Malfunction Due to the Needle Guard Coming Off During Ventriculoperitoneal Shunt Surgery

**DOI:** 10.7759/cureus.72228

**Published:** 2024-10-23

**Authors:** Hajime Ono, Tomohiro Kaji, Hiroyuki Morishima, Goro Nagashima

**Affiliations:** 1 Neurosurgery, Kawasaki Municipal Tama Hospital, Kawasaki, JPN

**Keywords:** needle guard, patient safety, shunt malfunction, shunt valve, ventriculoperitoneal shunt surgery

## Abstract

We report a rare case of shunt valve failure due to obstruction during ventriculoperitoneal (VP) shunt surgery for hydrocephalus after subarachnoid hemorrhage due to aneurysm rupture. The hydrocephalus shunt surgery was started normally, and there was no bending or twisting of the valve, nor blood contamination. However, after irrigation of the shunt valve, the shunt valve obstructed and malfunctioned before catheter connection and insertion into the subcutaneous space. Shunt valves are rarely damaged during surgery. In this case, the cause of the malfunction could not be identified during surgery, and it was necessary to use a shunt valve made by another company for patient safety. The surgery was completed without incident, but the cause of the obstruction, which was discovered after surgery, was that the needle guard inside the valve had come off from the bottom. The CODMAN CERTAS Plus Programmable Valve (CCPPV) in particular has excellent functionality, but the regular type needle guard is attached to the bottom of the valve pump. Therefore, it cannot withstand the handling during surgery that other valves tolerate. In the future, improvements in medical equipment and more careful operation of shunt valves by surgeons are required for risk management during surgery.

## Introduction

Ventriculoperitoneal (VP) shunt surgery for hydrocephalus is known to be associated with a variety of problems and complications. Shunt system mechanical failure occurs most frequently as a result of either catheter or valve obstruction. Many mechanical problems are caused by shunt valves becoming blocked because of blood mixing, disconnection, migration, intracranial catheter obstruction, or malposition [1]. Complications with the valve reservoir or flushing device have been noted [2], but reports of complications with the pressure valve itself are rare. Although many improvements have been made to shunt valves, they remain very delicate and precise, and thus, surgeons should be careful when handling them during placement. In addition to making changes in shunt devices, the handling of shunts during placement should be more widely publicized. The present case report describes a patient who required emergency replacement of a CODMAN CERTAS Plus Programmable Valve (CCPPV) shunt valve after the needle guard of the reservoir detached from the flat bottom.

## Case presentation

**Figure 1:**
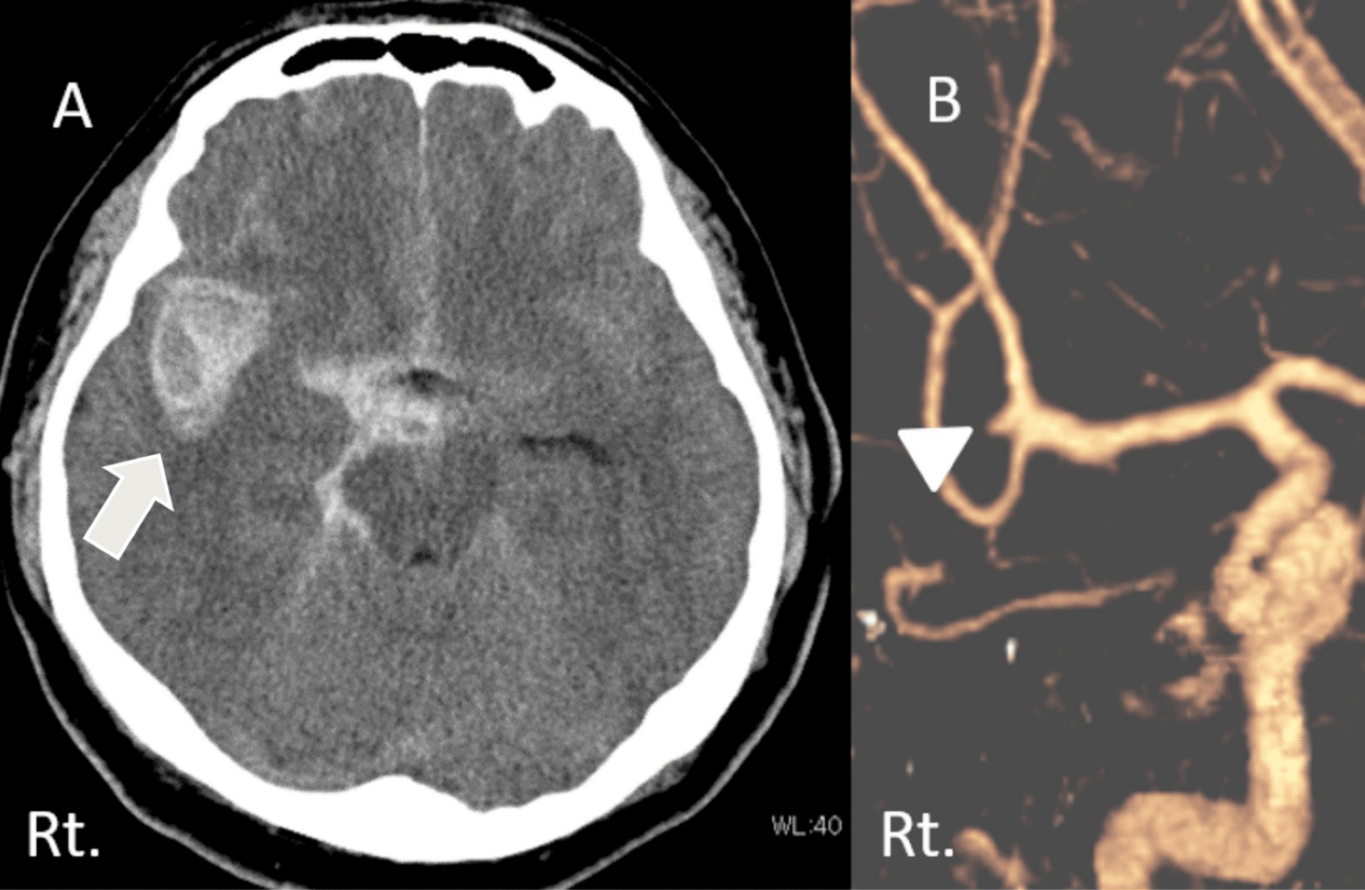
Head CT scan on admission (A) and CT angiography (B) A: Head CT scan showing subarachnoid hemorrhage and right intracerebral hemorrhage (white arrow). B: Contrast CT scan showed a ruptured aneurysm at the bifurcation of the right middle cerebral artery (white arrowhead).

**Figure 2:**
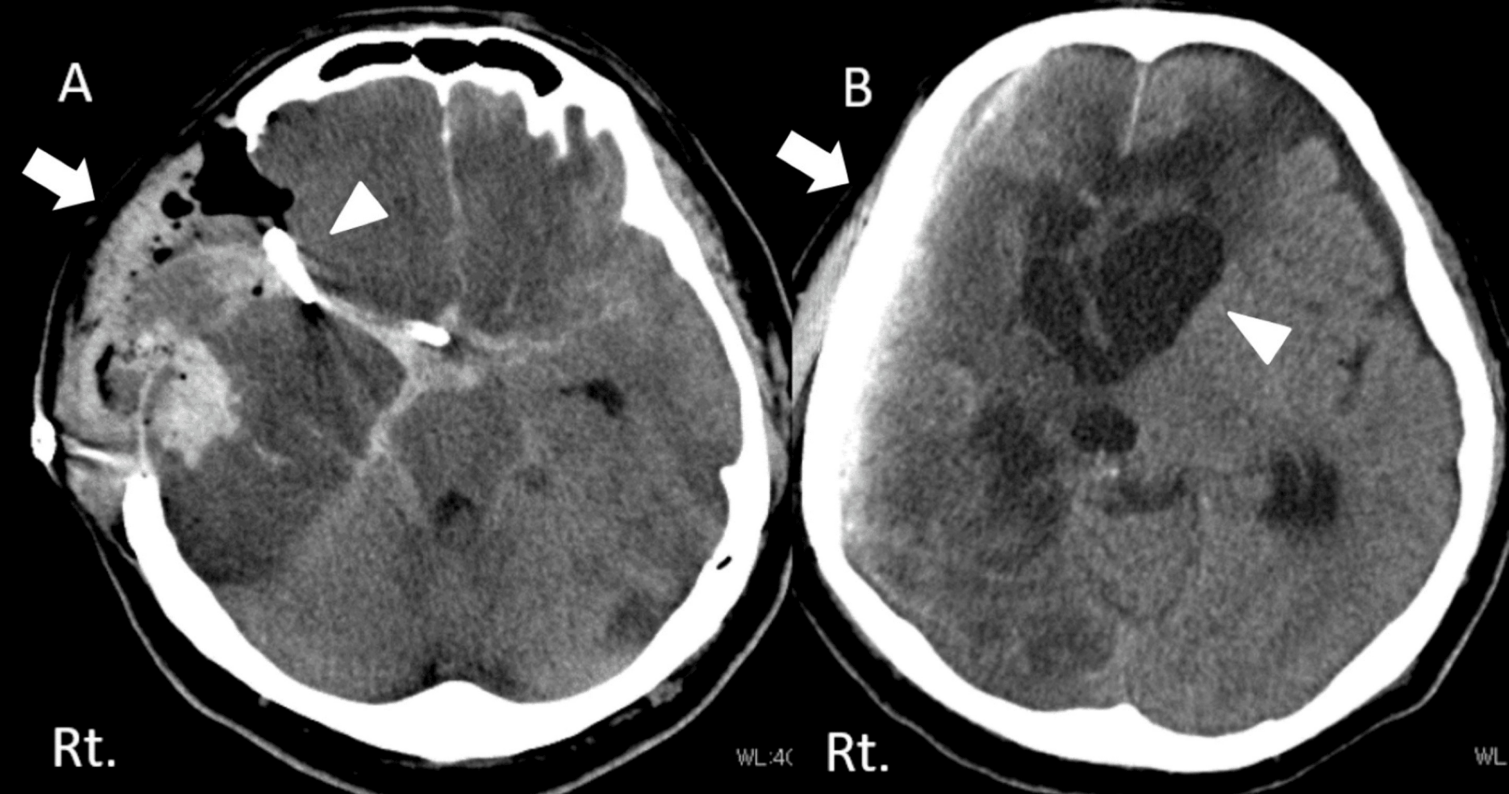
Postoperative CT scan post clipping (A) and post-cranioplasty (B) A: Emergency external decompression surgery was performed with craniotomy clipping (white arrow) and hematoma removal (white arrow) on the right side. B: Head CT scan of the head shows artificial bone cranioplasty (white arrow) and enlargement of the anterior horns (white arrowhead), suggestive of hydrocephalus.

**Figure 3:**
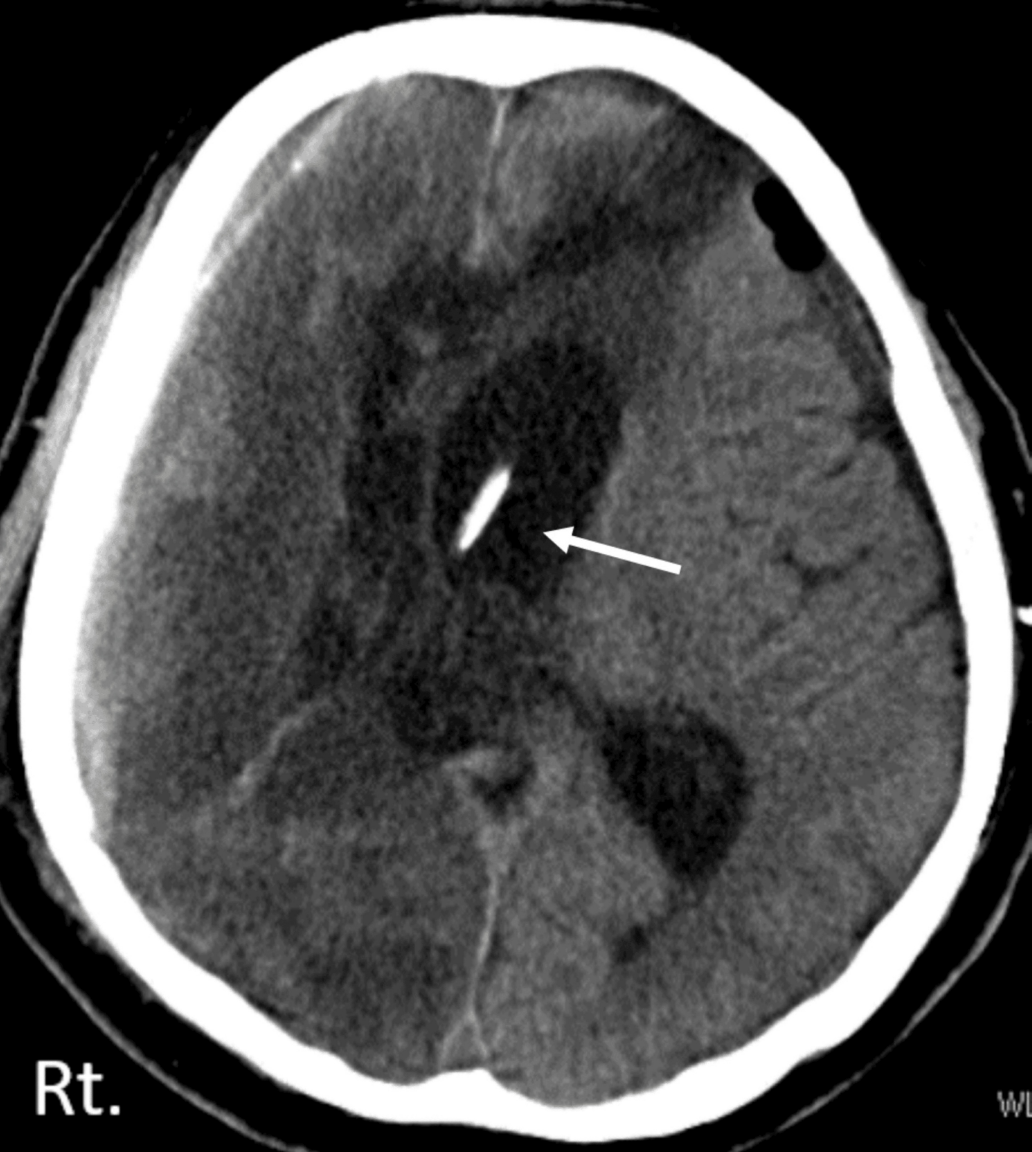
Head CT post-VP shunt surgery Head CT scan showed the tip of the ventricular shunt catheter in the left anterior horn (narrow white arrow).

The patient was a 54-year-old left-handed man. His only medical history was dyslipidemia. In April 2023, his family discovered him at home with impaired consciousness and requested emergency transport to our hospital. On admission, his vital signs were blood pressure of 210/100, heart rate of 65 beats/minute, and respiratory rate of 20 breaths/minute. Neurological findings showed a consciousness level of E2V1M4 on the Glasgow Coma Scale (GCS). In addition, he had a large right pupil (diameter: 6 mm) with anisocoria and severe left hemiplegia. After stabilization of his vitals, a head CT scan was performed and showed diffuse subarachnoid hemorrhage with right intracerebral hemorrhage (Figure 1A). Furthermore, contrast CT revealed that the cause was a right middle cerebral artery aneurysm, and the diagnosis was severe subarachnoid hemorrhage due to a right middle cerebral artery aneurysm rupture (Figure 1B). As a result, emergency external decompression surgery was performed along with right craniotomy clipping and hematoma removal (Figure 2A). Intracranial pressure management was still required, and intensive care, including respiratory management, was performed in the ICU. One month later, a right cranioplasty using artificial bone was performed (Figure 2B), and the patient's level of consciousness improved (GCS: E3V1M6). Moreover, one month later, the patient's level of consciousness decreased due to hydrocephalus following subarachnoid hemorrhage, and the decision was made to perform a left VP shunt (Figure 3).

Operative findings and cause of shunt malfunction

**Figure 4:**
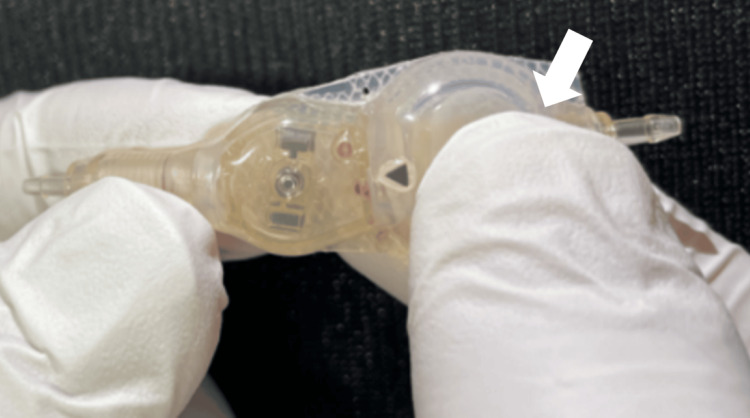
Operative view of pumping the CCPPV To check the discharge of the valve to be installed, a saline-filled reservoir was placed between the fingers and pumped (white arrow). CCPPV: CODMAN CERTAS Plus Programmable Valve

In our facility, we usually use the CCPPV for hydrocephalus shunt surgery. VP shunt surgery was started under general anesthesia using the CCPPV shunt system. Incisions were made on the cranial and ventral sides, and the shunt valve was set on the operating table. As usual, the priming connector was attached to the valve, and a syringe filled with saline was used to pump water across the valve to remove any air. To check the valve discharge again, the saline-filled reservoir was held between the fingers and pumped (Figure 4). Next, the valve and ventricular catheter were fixed with nylon thread, the catheter was re-immersed in saline to check for blockages, and pumping was performed to check for drainage; however, drainage was found to be insufficient. After the catheter had been placed and secured, drainage was still insufficient, even when the reservoir was pressed with a finger, leading the surgeons to consider whether there was a problem with the catheter fixation. Therefore, the fixed nylon thread was removed, and the pumping part was pressed into a container filled with saline. However, the pumping part did not return sufficiently like a check valve, and drainage was also insufficient. During the operation, we determined that the valve itself was defective. Therefore, we urgently switched to a valve manufactured by another company and successfully completed the operation.

**Figure 5:**
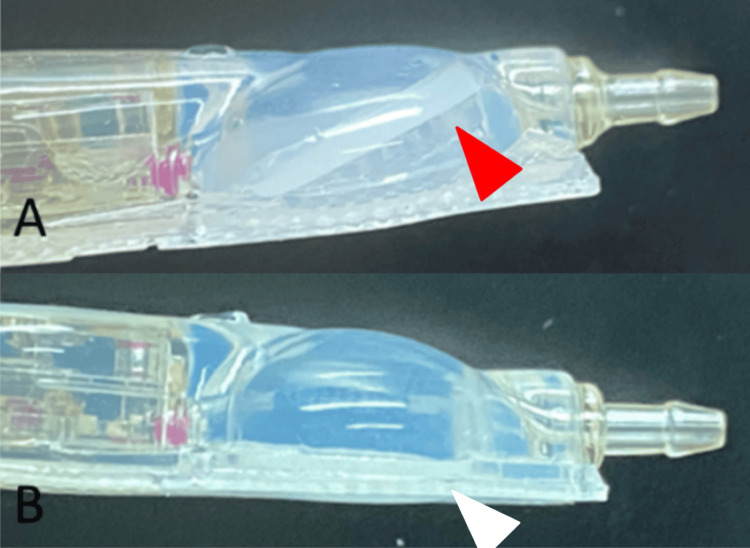
Damaged CCPPV (A) and normal CCPPV (B) A: CCPPV with shunt dysfunction: There was no blood contamination or external damage, but the needle guard attached to the bottom of the reservoir had come off (red arrowhead). B: Normal CCPPV: In a normal structure, the needle guard is not detached from the reservoir (white arrowhead). CCPPV: CODMAN CERTAS Plus Programmable Valve

After the operation, we searched for the cause of the shunt malfunction and confirmed the CCPPV. During the operation, the surgeon operated the CCPPV shunt system using normal procedures. The CCPPV shunt valve was operated without bending or twisting, and water was passed through it, and the catheter was connected. However, when the troubled CCPPV was checked after the operation, it was found to have a different structure than normal. There was no blood contamination or external damage, but the needle guard attached to the bottom of the reservoir had peeled off (Figure 5). In other words, it was discovered that the needle guard had peeled off from the bottom inside the CCPPV valve, causing the shunt malfunction.

## Discussion

In Japan, VP shunt surgery is a very common surgical option for hydrocephalus treatment. In addition, depending on the patient's age, disease, and condition, endoscopic surgery or other options may be selected [3]. In our medical institution, VP shunt surgery is the basic surgical treatment for hydrocephalus after subarachnoid hemorrhage, as in this case [4]. We use CCPPV for the shunt valve because it allows for fine pressure changes compared to other shunt valves and is effective in the postoperative management of hydrocephalus [5-7].

On the other hand, shunt surgery involves placing a foreign body in the body, and there is debate about postoperative complications. In particular, it is well known that the main causes of shunt dysfunction are obstruction and infection [8,9], and careful technique is required in shunt surgery [10]. However, damage to the shunt valve during surgery in this case is extremely rare, and a search of the literature database did not reveal any similar reported cases. Therefore, we report the handling method of the shunt valve and surgical management.

Firstly, the structure of the shunt valve should be considered. Broadly classified, CCPPV has two sizes (standard and small). In both sizes, the anti-siphon device and the variable valve are integrated. There is a difference in the needle guard between the two sizes. The needle guard is integrated in the shunt valve in the small type, but not in the standard type. In the standard type, the needle guard is simply attached.

Secondly, the surgeon's operation during surgery should be discussed. The surgeon operated the CCPPV shunt valve without bending or twisting it and connected the catheter. However, in this case, a regular type CCPPV was used in which the valve and needle guard were not integrated. Of course, the possibility of valve damage before surgery is extremely low. Furthermore, a needle guard that is integrated with the bottom of the reservoir is not likely to come off under stress. Therefore, if the cause of the shunt malfunction in this case is the coming off of the needle guard, it is necessary to pump to check the discharge of cerebrospinal fluid after the catheter is attached [11]. Pumping was performed by pinching the valve with fingers. If pinching the valve with fingers leads to the coming off of the needle guard, the surgeon's operation can also be cited as one of the causes of the malfunction. When the CCPPV became blocked, it was necessary to change the shunt valve. At that time, there were opinions in favor of using a backup CCPPV. However, since the cause of the blockage was unknown, a shunt valve made by another company was necessary for the success of the operation. Then, we replaced the shunt valve, connected the catheter, and inserted the system into the subcutaneous space. Although changing the shunt system during the operation was an unexpected event, it was necessary not only for the success of the shunt operation but also for medical safety management for the patient.

Thirdly, we will present some precautions regarding the handling of CCPPV. One of the ways to handle the shunt valve is "how to pass water through the CCPPV shunt valve." When handling the shunt valve, when passing water through the CCPPV shunt valve, (1) care should be taken not to press the reservoir too hard or apply pressure when passing water through. (2) A priming connector should be used when filling with saline. (3) It seems that if the valve is damaged or bent, the silicone housing may be damaged, the needle guard may come off, or the shunt system may be blocked. The small-sized CCPPV has an integrated needle guard, which is stronger than the standard type and is safe to handle.

Fourthly, we would like to discuss future issues. According to the Japanese manufacturer, the standard CCPPV is used in many cases in Japan. The manufacturer seems to be aware of some individual reports of needle guards peeling off, but the exact incidence rate has not been made public. Furthermore, other manufacturers' data management is similar. Therefore, even with other shunt valves, if they are equipped with a siphon guard, pumping may be required to check drainage [11]. Also, it is important to note that pumping to drainage does not require strong force from the surgeon.

**Figure 6:**
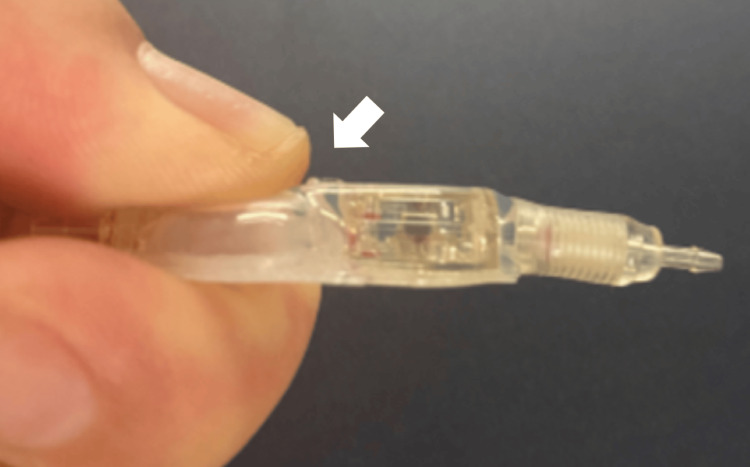
Not recommended ways to hold the valve Bending the regular CCPPV reservoir or pinching it between your fingers may cause the needle guard to peel off easily (white arrow). CCPPV: CODMAN CERTAS Plus Programmable Valve

In other words, if failures such as this case are not feed back to the surgeon, there is a possibility of trouble even if standard procedures are followed. In addition, the stress of the CCPPV valve is not recognized, but it is probably different from other valves. In other words, when pumping to check water flow, it is necessary to operate on a flat surface such as the skull where the shunt valve is installed, and it is better to refrain from operations such as pinching the CCPPV valve tightly with the surgeon's fingers (Figure 6).

Also, after connecting the shunt valve to the ventricular catheter, surgeons may use a shunt pump to ensure that cerebrospinal fluid is flowing properly before placing the shunt system subcutaneously, so it remains unclear how to prevent failure during such procedures. In this case, the needle guard was peeled off due to normal pumping operation, so if the same CCPPV is selected in the future, it would be better to use a small model with an integrated needle guard [12]. In any case, it is necessary to strengthen the adhesion during the manufacturing process of the regular model and perform careful pumping operations.

Finally, shunt surgery is common in the field of neurosurgery and is a procedure that can achieve high therapeutic effects with standardized methods. We look forward to publication and feedback in the near future, including improvements to the shunt valve and consideration of the causes of damage.

## Conclusions

It is unlikely that the standard CCPPV shunt valve used in this case did not meet the manufacturer's specifications when it was shipped from the manufacturing plant. However, it is believed that an unforeseen incident occurred due to the surgeon's handling and technique during surgery, leading to the blockage. VP shunt surgery for hydrocephalus is a common surgery in the field of neurosurgery. Therefore, there is no need to change the surgical method performed at each facility. However, to minimize the risk of damage to CCPPV during surgery, it is necessary to handle the valve gently and without bending it. In this report, we presented the need for surgeons to be aware of malfunctions as well as handling and precautions for CCPPV. At the same time, the role of surgeons in reporting equipment failures is also considered to be important in the future for improving medical devices.
